# Liver Graft Pathology and Low Serum 25-Hydroxyvitamin D after Living Donor Liver Transplantation

**DOI:** 10.3390/metabo12050388

**Published:** 2022-04-25

**Authors:** Shu-Hsien Lin, Chih-Chi Wang, Kuang-Tzu Huang, Kuang-Den Chen, Li-Wen Hsu, Hock-Liew Eng, King-Wah Chiu

**Affiliations:** 1 Division of Hepato-Gastroenterology, Department of Internal Medicine, Kaohsiung Chang Gung Memorial Hospital, Kaohsiung 83301, Taiwan; linsusan77628@gmail.com; 2 Liver Transplantation Program, Kaohsiung Chang Gung Memorial Hospital, Kaohsiung 83301, Taiwan; huangkt@cgmh.org.tw (K.-T.H.); dennis8857@gmail.com (K.-D.C.); hsuliwen@ms55.hinet.net (L.-W.H.); 3 Division of General Surgery, Department of Surgery, Kaohsiung Chang Gung Memorial Hospital, Kaohsiung 83301, Taiwan; 4 College of Medicine, Chang Gung University, Taoyuan 33302, Taiwan; eng6166@gmail.com; 5 Institute for Translational Research in Biomedicine, Kaohsiung Chang Gung Memorial Hospital, Kaohsiung 83301, Taiwan; 6 Department of Pathology, Kaohsiung Chang Gung Memorial Hospital, Kaohsiung 83301, Taiwan

**Keywords:** acute rejection, CYP2R1, liver pathology, serum 25(OH)D, vitamin D receptor

## Abstract

Background: Most cases of advanced liver diseases are associated with low serum 25-hydroxyvitamin D and vitamin D deficiency. This phenomenon may occur in living donor liver transplantation (LDLT). Aims: We conducted this study to explore the interplay between VDR and CYP2R1 in liver graft and compared our findings with the pathological interpretation of serum 25(OH)D concentration. Methods: In total, 60 patients received liver graft biopsy after LDLT and were separated (1:1) into two groups: graft rejection group and graft non-rejection group. We extracted both of the recipients’ and donors’ serum DNA to investigate the vitamin D receptor (VDR) rs2228530 and CYP2R1 rs10741657 single nucleotide polymorphisms (SNPs) using real-time polymerase chain reaction. We also extracted DNA from liver graft tissues to explore the genetic alleles of VDR rs2228530 and CYP2R1 rs10741657 after LDLT. Serum biochemistry profile and 25(OH)D concentrations were measured before and after LDLT. Results: There were no significant differences in serum VDR rs2228530 and CYP2R1 rs10741657 genetic alleles between recipients and donors. The percentage of genetic modification was 33.4% (10/30) for the rejection and non-rejection groups in VDR rs2228530, and 66.7% (20/30) for both groups in CYP2R1 rs10741657. Serum 25(OH)D concentrations were significantly lower after LDLT D30 than that before LDLT in the rejection (*p* = 0.0001) and non-rejection graft pathology (*p* = 0.0017) groups. Conclusions: The presence of low serum 25(OH)D concentrations after LDLT suggested that post-transplant low serum 25(OH)D concentrations may develop with the homogenous phenomenon of VDR rs2228530 and CYP2R1 rs10741657 genetic modifications in recipients regardless of graft pathology.

## 1. Introduction

Because activated vitamin D is predominantly synthesized in the liver, low 25(OH)D is a predictor of and is closely associated with severe liver disease, particularly in cases with poor prognosis [1,2,3,4,5]. The prevalence of vitamin D deficiency is higher in hepatitis-C-related cirrhotic patients compared to non-cirrhotic patients and correlates with components of hepatic function [1]. In addition, Vitamin D deficiency was observed in the majority of HBV-infected patients and associated with adverse clinical outcomes [3]. According to previous study conducted by Barchetta, I., et al., low 25(OH)D levels are associated with the presence of non-alcoholic fatty liver disease (NAFLD) independently from metabolic syndrome, diabetes and insulin-resistance profile [5]. In literature, vitamin D has been reported to act as a regulator of both innate immunity and adaptive immunity, and it has significant association with liver allograft function, such as the incidence of acute rejection and infection during the first month of transplantation [6]. An interesting issue that warrants investigation in liver transplantation is when a new liver graft implant is provided to a patient with end-stage liver disease. According to our previous studies about cytochrome P450 in living donor liver transplantation (LDLT), a homogenous phenomenon could exist when the recipient and donor have a different genetic polymorphism [7,8,9,10]. Moreover, a liver graft with a different genotyping allele on vitamin D-related genetic polymorphism could be used. Recent studies have suggested that vitamin D receptor (VDR) and CYP2R1 may be closely related to the synthesis of vitamin D [11]. VDR is widely expressed in the liver and inflammatory cells of chronic liver disease patients and its expression is negatively associated with the severity of liver histology in both nonalcoholic steatohepatitis (NASH) and chronic hepatitis C (CHC) patients [11]. In this current study, we aimed to explore the association of low serum 25(OH)D concentration after LDLT with liver graft pathologic findings (acute rejection and non-rejection), and also to investigate the presence of homogenous phenomenon of VDR rs2228530 and CYP2R1 rs10741657 genetic modifications in these biopsied recipients in different liver graft pathologic change.

## 2. Results

### 2.1. Clinical Profiles and Liver Graft Pathological Interpretation of Study Population

Total 190 patients underwent LDLT in this 2-year research. There were 60 recipients enrolled in our study. 30 recipients developed post-LDLT acute rejection, therefore, the rejection rate was 15.8% (30/190). The mean age of the recipients in the rejection group and non-rejection group and the donors were 56.1, 55.7 and 34.6 years old, respectively. Genders (male/female) of the recipients in the rejection group and non-rejection group and the donors were *n* = 18/*n* = 12, *n* = 17/*n* = 13, and *n* = 29/*n* = 31, respectively. The underlying diseases were hepatitis B virus (HBV)-related liver diseases in 33 cases (included 11 cases with end stage liver diseases (ESLD) and 22 with hepatomas); hepatitis C virus (HCV)-related ESLD in 9 cases (included 6 with hepatomas and 1 HBV plus HCV); alcoholic liver disease in 9 cases; primary biliary cirrhosis in 3 cases; and cryptogenic cirrhosis in 6 cases. In addition, recipients who did not undergo liver graft biopsy (*n* = 30) were also evaluated as normal control group in this study. The mean age of the recipients in the normal control group was 57.6 years old. There were 16 males and 14 females. The primary underlying liver diseases were HBV-related liver diseases in 13 cases (included 6 cases with ESLD and 7 with hepatomas); HCV-related liver diseases in 14 cases (included 5 cases with ESLD and 19 with hepatomas); alcoholic liver disease in 2 cases, and cryptogenic cirrhosis in 1 case. The mean graft cold ischemic time and warm ischemic time were 51.9 ± 64.6 min and 37.9 ± 6.7 min. Graft-to-recipient-weight ratio was >0.8%. Recipients with unexplained abnormal liver functions as clinically required will undergo graft biopsy to evaluate etiology according to pathology result. In the rejection group, 76.7% (23/30) of patients had mild degree acute rejection, and 20% (6/30) had moderate degree acute rejection (Table 1). One case was defined as early chronic rejection and received re-transplantation 2 months later.

In the non-rejection graft pathology group, 40.0% (12/30) had fatty liver disease, including 20.0% (6/30) with mild degree (<5%), 6.7% (2/30) with moderate degree (6–20%), and 13.3% (4/30) with severe degree (>2%). All of those donors were within normal limits for body mass index and had not been diagnosed with a non-alcoholic fatty liver disease during donor evaluation before LDLT. Notably, 20.0% (6/30) of patients had recurrent hepatitis C infection who had not received directed active anti-viral agent or inferno plus ribavirin treatment before LDLT. Furthermore, 20.0% (6/30) of patients were diagnosed with a non-specific reactive change during the study. One case was diagnosed with a post-transplantation lymphoproliferative disorder and survived after optimal chemotherapy.

### 2.2. Genetic Polymorphisms of VDR rs2228530 and CYP2R1 rs10741657

The serum study on recipients and donors of the VDR rs2228530 and CYP2R1 rs10741657 is shown in Table 2. The genotyping allele frequencies were greater in AG and GG alleles and were smaller in AA alleles between the recipients and donors before LDLT, but these differences were not significant (*p* = 0.414). By contrast, in CYP2R1 rs10741657, the genotyping allele frequencies were greater in AA and GG alleles and smaller in AG alleles between the recipients and donors before LDLT, but this different was also not significant (*p* = 0.143).

### 2.3. Modification of VDR and CYP2R1 Genetic Polymorphisms after LDLT

In the liver graft biopsy tissues study (Table 3), the VDR rs2228530 genotyping allele was modified in 33.4% (10/30) of patients; 20% had GG to AG, 6.7% had AA to AG, and 6.7% had AG to GG changes in the rejection group, and in the non-rejection graft pathology group, 20% had GG to AG, and 13.3% had AA to AG modifications (*p* value = 0.371). By contrast, 66.7% (20/30) of patients did not have modified genotyping, with 53.3% AG and 13.3% GG alleles in the rejection group, and 40% AG, 20% GG, and 6.7% AA alleles in the non-rejection graft pathology group (*p* = 0.260).

For the modifications of CYP2R1 rs10741657 genotyping after liver graft biopsy, 66.7% (20/30) of patients showed modification in both rejection groups, including 20% with GG to AA, 20% with AA to AG, 13.3% with GG to AG, 6.7% with AG to AA, and 6.7% with AG to GG modifications in the rejection group, and in the non-rejection graft pathology group, 33.3% with GG to AA, 13.3% with GG to AG, 13.3% with AA to AG, and 6.7 with AA to GG modifications (*p* = 0.212). On the other hand, only 33.4% of patients did not have genetic allele modification in both groups, including 26.7% for AG and 6.7% for AA alleles in the rejection group, and 26.7% for AG and 6.7% for GG alleles in the non-rejection graft pathology group (*p* = 0.226).

### 2.4. Serum 25(OH) Concentration and Biochemistry Profile

As a normal control, we conducted the post LDLT day-30 (D30) serum 25(OH)D level in the non-biopsy group of recipients (*n* = 30). The mean serum 25(OH)D level was 44.59 ± 36.93 ng/mL, which was statistically significantly higher than both rejection (11.01 ± 7.64, *p* < 0.005) and non-rejection with graft pathology groups (11.32 ± 7.55, *p* < 0.005) after LDLT. In the graft rejection group, low serum 25(OH)D concentrations were significantly lower after LDLT compared with before (*p* = 0.0001). The same was also true after LDLT (*p* = 0.0017) in the non-rejection graft pathology group (Table 4).

## 3. Discussion

Serum 25(OH)D concentrations have been suggested as predictors for advance end-stage liver disease and liver cirrhosis with severe fibrosis, particularly in cases of non-alcoholic steatohepatitis or chronic C hepatitis infection [1,3,5]. In the current study, there were only nine cases with chronic C hepatitis, including six cases associated with hepatocellular carcinoma. For patients with vitamin D deficiency, recent studies have suggested that a genetic relationship between patients with VDR and CYP2R1 gene variants may be linked to the deficiency [11,12]. Our recent study showed that VDR rs2228530 genetic alleles with AA and AG may be associated with the development of non-alcoholic fatty liver disease after LDLT [13]. In the current study, the gene variations in the allelic frequency of AA, AG, and GG were not significantly different in VDR rs2228530 and CYP2R1 rs10741657 in the recipients and donors before LDLT. It is notable that genetic polymorphic modification exists in the liver graft tissues not only in the VDR rs2228530 genotyping alleles but also in the CYP2R1 rs10741657 SNP, with up to eight allele components (GG to AA, AA to AG, GG to AG, AG to AA, AG to GG, and AA to GG) modified. In our previous studies of cytochrome P450 in LDLT, a homogenous phenomenon might be emphasized here; there were many genetic polymorphisms, such as CYP2C19, CYP3A4, CYP3A5, MDR-1, and IL-28B, with complicated modifications after LDLT [8,14]. This phenomenon will be stabilized as time goes by, particularly 1 month after LDLT, because acute rejection is common within 1 month after LDLT [15]. All of the modified genotypes stabilized 1–11 months after LDLT, as proved by biopsy of liver tissues [10]. A recent study has also suggested that serum concentrations [16] of 25(OH)D and vitamin D bounding protein should rise substantially following transplantation [17]. In our current results, low serum 25(OH)D was observed both in the liver graft rejection and non-rejection graft pathology groups. This finding may represent the acute phase of unstable cytochrome P450 modification with two variant genes in the liver graft after LDLT.

As shown in Table 3, the VDR rs2228530 genotyping allele was modified in 33.4% (10/30) of recipients in both rejection and non-rejection groups; the modifications of CYP2R1 rs10741657 genotyping was 66.7% (20/30) in both rejection and non-rejection groups, respectively. Although genetic modifications of VDR rs2228530 and CYP2R1 rs10741657 may be associated to low serum 25(OH)D concentrations, there was no significant association between liver graft rejection and these changes of SNPs. In our current study, we already directly examined the correlation of final genetic polymorphisms and genetic modification of liver graft with serum 25(OH)D level. There was no statistically significant difference between all of the sub-groups of genetic polymorphisms, as shown in Table 3, which may be due to the small cases number in each subgroup of the genetic polymorphisms in VDR and CYP2R1. However, low serum 25(OH)D levels were found in both groups, regardless of rejection. On the other hand, we would like to put more emphasis on exploring the association between liver graft pathology even in acute rejection or non-rejection and the low serum 25(OH)D after LDLT. The current results suggested that the presence of low serum 25(OH)D concentrations were found in both acute rejection and non-rejection with graft pathology groups after LDLT.

The association of vitamin D deficiency with disease severity, infection, and worse prognosis was previously reported in patients with liver disease. For liver transplant recipients, preoperative serum vitamin D status was correlated with disease severity and highly associated with invasive infection in the first 28 post-transplant days (PODs). Furthermore, the value on POD 28 had a strong association with graft function [18]. In Table 4, serum 25(OH)D levels of recipients before transplantation were within the normal range, which might be explained for the reason that nearly half (*n* = 28/60) of the recipients enrolled in this study were transplanted with the indication of hepatomas rather than that of acute decompensated liver cirrhosis or acute liver failure; thus, these recipients had pre-LDLT normal serum 25(OH)D levels due to relatively preserved liver function. In our study group, 6 of the 28 hepatomas developed in an HCV-related ESLD conditions; of course, these 6 recipients also presented reduced serum 25(OH)D levels. It is well-established in the literature that hepatomas are associated with reduced 25(OH)D serum levels, mainly because most of them developed in a cirrhotic/fibrotic liver. Actually, all of the remaining 22 hepatomas of our series developed in a non-cirrhotic/fibrotic liver. 

According to a previous report, vitamin D deficiency correlated with liver insufficiency, and 25(OH)D concentration would significantly improve after liver transplantation; however, still more than 50% of recipients were significantly deficient after liver transplantation [19]. In our liver transplantation program, all recipients received the post-transplant immunosuppressants treatment protocol. There was no protocol liver graft biopsy after LDLT. Only those who had graft dysfunction would undergo liver graft biopsy for being suspicious of acute rejection clinically. Although many factors such as infection or post-transplant treatments (immunosuppressants, chemotherapy …) would potentially affect 25(OH)D serum levels, the pathology findings were regarded to be the gold standard for determining the leading cause of graft dysfunction, and this histopathologic change might be the most significant factor associated to post-LDLT low 25(OH)D serum levels in these biopsied recipients (*n* = 60) in our study. 

In our current study, all of the 60 recipients who underwent biopsy had abnormal graft function. The timing at which the serum 25(OHD) concentration was measured prior to LDLT and after LDLT was the same as the timing at which the liver biopsy was performed. Notably, their serum 25(OH)D concentrations were significantly lower after LDLT than before LDLT, regardless of liver graft pathology. Moreover, in recipients who did not undergo liver graft biopsy because of normal liver function, we additionally examined the post-LDLT D30 serum 25(OH)D level in the non-biopsy group of recipients as a normal control. The post-LDLT D30 serum mean 25(OH)D level in the non-biopsy group of recipients was statistically significantly higher than both rejection and non-rejection with graft pathology groups after LDLT. The result suggests that normal liver graft function will not cause the decrease of serum 25(OH)D level.

Evidence from the research of Zhou Q et al. suggests that vitamin D deficiency is an important independent risk factor for acute rejection; vitamin D supplementation may reduce the risk of acute cellular rejection and infection in vitamin D deficient liver allograft recipients [6]. The mechanisms of the protective role of 25(OH)D for the allograft are as follows: VDR is ubiquitously expressed in immune cells, including T lymphocyte and cells of innate immune system [16]. Vitamin D supplementation increases Treg cells and T memory cells and is associated with a lower risk of acute rejection and infection, suggesting vitamin D as playing the role of immune modulator through inducing Treg cell differentiation [6].

In this study, low serum 25(OH)D levels after LDLT were measured in recipients undergoing graft biopsy for the reason of post-transplant abnormal liver function, and the low 25(OH)D levels in serum after LDLT were observed in rejection and non-rejection groups (including fatty change, acute cholangitis, recurrent hepatitis, non-specific pathological change and PTLD in pathology) (Table 1). Furthermore, we figured out that serum low 25(OH)D concentrations developed in the enrolled recipients who have homogenous phenomenon of genetic modifications of VDR rs2228530 and CYP2R1 rs10741657 polymorphisms.

There are some limitations in our current study. Because protocol liver biopsy after LDLT is not permitted in our liver transplant program, we only evaluated 60 recipients who had received liver graft biopsy as clinically required for evaluation of post-LDLT abnormal liver function in our liver transplantation program.

The strength of our current study is the clinical findings following our previous research, firstly suggesting the homogenous phenomenon of sequence changes in graft liver CYP2C19 from the different genotypes between the donors and the recipients [9], and then further observing an association between subclinical low serum 25(OH)D in donors and fatty liver disease in recipients after LDLT [13]. Based on the result of these two previous studies, we reported the correlation that the serum vitamin D level was significant lower in cases with genetic modification than those with non-modification [9,13,20]. This study demonstrated that the percentage of genetic modification was 33.4% (10/30) for the rejection and non-rejection groups in VDR rs2228530, and 66.7% (20/30) for both groups in CYP2R1 rs10741657. Serum 25(OH)D concentrations were significantly lower between before and 30-days after LDLT, compared with before LDLT, in the rejection (*p* = 0.0001) and non-rejection graft pathology (*p* = 0.0017) groups.

In conclusion, we would like to emphasize that the development of low serum 25(OH)D concentrations occurred after LDLT in both acute rejection and non-rejection group with abnormal liver function and graft pathology such as fatty change, recurrent hepatitis, acute cholangitis, non-specific pathological change and PTLD. Additionally, from the viewpoint of genetic variants in the VDR gene for regulating vitamin D levels, this finding also suggested that post-LDLT serum low 25(OH)D level would occur in recipients with homogenous phenomenon of genetic modifications of VDR rs2228530 and CYP2R1 rs10741657 polymorphisms, regardless of graft pathology.

## 4. Patients and Methods

### 4.1. Study Population

In this 2-year research period, there were 190 patients receiving liver transplantation in our hospital. The exclusion criteria were acute issues on chronic liver failure due to sepsis, primary bacterial peritonitis, esophageal variceal bleeding with shock *(n* = 108), biliary atresia (*n* = 21), pediatric LDLT due to glycogen storage disease, Wilson’s disease (*n* = 16), and diseased donor liver transplantation (*n* = 6). The inclusion criteria were an adult liver transplantation who received a selective post-LDLT liver graft biopsy as clinically required. Finally, a total of 60 recipients was enrolled in our study. In addition, recipients who did not undergo liver graft biopsy (*n* = 30) were also evaluated in this study as normal control group. 

Because protocol liver biopsy after LDLT was not permitted in our liver transplant program, all of the enrolled cases underwent liver graft biopsy for clinically being suspicious of acute rejection-related graft dysfunction or to evaluate the causes resulting in post-LDLT abnormal liver function, defining as biochemistry test with “one” or “both” of the following condition: (1) elevated AST and ALT tripled than normal level (2) Total bilirubin level > 2 mg/dL without evidence of biliary complication. All of the liver graft biopsy procedures were performed 1–4 weeks after LDLT. The biopsy tissue was analyzed by two independent liver transplant pathologists. Depending on the histological diagnosis, the patients were divided into two groups: graft rejection group (*n* = 30), and graft non-rejection group (*n* = 30).

### 4.2. Laboratory Assessment

#### 4.2.1. Serum 25(OH)D and Serum Biochemistry

Serum 25(OH)D concentrations were measured in recipients before and after LDLT 30-day (D30) and the same time of graft biopsy by use of a 25(OH) vitamin D enzyme-linked immunosorbent assay kit (Enzo Life Sciences Inc., Farmingdale, NY, USA). Serum biochemistry profiles were measured after LDLT with the same timing at which the liver biopsy was performed. We have additionally examined the post-LDLT D30 serum 25(OH)D level in the non-biopsy group of recipients (*n* = 30) as a normal control. The optical density of each well at 405 nm was determined within 30 min with a microplate reader. Each sample was assayed in duplicate by a single operator to assess interassay precision. By definition, serum 25(OH)D levels of greater than or equal to 30 ng/mL (75 nmol/L) were considered within normal limits in our study.

#### 4.2.2. VDR rs2228530 and CYP2R1 rs10741657

Genomic DNA was extracted from the peripheral blood mononuclear cells of donors and recipients before LDLT by use of a QIAamp DNA Blood Mini Kit (Qiagen, Hilden, Germany). Genotyping was performed to detect the single nucleotide polymorphisms (SNP) VDR rs2228530 and CYP2R1 rs10741657 with a ready-to-use, manufacturer-validated, predesigned allele-discriminating TaqMan SNP assay for polymerase chain reaction (PCR) amplification in clear optical 96-well plates on a 7500 Fast Real-Time PCR system (Applied Biosystems International, Foster City, CA, USA), according to the manufacturer’s instructions. The VDR SNP rs2228570 and CYP2R1 rs10741657 genotypes were also studied in liver tissues via selective graft biopsies after LDLT on the same 7500 Fast Real-Time PCR System with Custom TaqMan SNP genotyping assays (Applied Biosystems) for allele discrimination. Both the serum and liver graft biopsy tissue SNPs were selected according to VDR rs2228530 AA/AG/GG and CYP2R1 rs10741657 AA/AG/GG allele frequencies and functional clinical implications. All genotypes were assayed in duplicate to assess intraassay precision. Genetic modification is defined as the difference of the SNP of VDR rs2228570 and CYP2R1 rs10741657 genotypes before and after LDLT.

### 4.3. Ethics

The study was approved and authorized by the hospital’s ethical committee (Chang Gung Memorial Hospital of Taiwan; ethical approval number: 202001900A3). Our research was performed in accordance with relevant guidelines and regulations. Informed consent was obtained from all participants or their legal guardians in accordance with the Declaration of Helsinki. All of liver tissue in our study were procured from the liver transplantation center in Kaohsiung Chang Gung Memorial Hospital. No liver tissues were procured from prisoners.

### 4.4. Statistics

Statistical analyses were performed using SPSS statistical software (SPSS for Windows, version 14.0; Chicago, IL, USA). Fisher’s exact test with a two-sided model was used to compare VDR rs2228530 and CYP2R1 rs10741657 genetic polymorphisms in donors and recipients before LDLT, and the SNP allele modifications in VDR rs2228530 and CYP2R1 rs10741657 genotypes of the liver graft biopsy tissues. Student’s *t*-tests were used to compare serum 25(OH)D concentrations in the rejection and non-rejection graft pathology groups before and after LDLT. *P* values less than 0.05 were considered to indicate a statistically significant difference.

## Figures and Tables

**Table 1:** Liver graft pathologies of rejection (*n* = 30) and non-rejection (*n* = 30) in the recipients who underwent living donor liver transplantation.

Category	Liver Graft Pathology	Episodes (%)
Rejection		
	Acute, mild	23 (76.7)
	Acute, moderate	6 (20.0)
	Chronic, early	1 (3.3)
Non-rejection		
	Fatty, mild	6 (20.0)
	Fatty, moderate	2 (6.7)
	Fatty, severe	4 (13.3)
	Acute cholangitis	5 (16.7)
	Hepatitis C recurrence	6 (20.0)
	Non-specific reactive change	6 (20.0)
	Post-transplantation Lymphoproliferative disorder	1 (3.3)

**Table 2:** Single nucleotide polymorphisms of vitamin D receptor and CYP2R1 in 60 pairs of recipients and donors who underwent living donor liver transplantation.

Genetic Alleles	Recipient, *n* (%)	Donor, *n* (%)	*p* Value
VDR rs2228530			0.414
AA	8 (13.3)	14 (23.3)	
AG	30 (50.0)	26 (43.3)	
GG	22 (36.7)	20 (33.3)	
CYP2R1 rs10741657			0.143
AA	14 (23.3)	8 (13.3)	
AG	20 (33.3)	30 (50.0)	
GG	26 (43.3)	22 (36.7)	

VDR: vitamin D receptor; Fisher’s exact test (two-sided).

**Table 3:** Modification of the vitamin D receptor and CYP2R1 genetic polymorphisms in recipients with graft rejection (*n* = 30) and non-rejection graft (*n* = 30) pathology after living donor liver transplantation.

Genetic Modified	Rejection, *n* (%)	Non-Rejection, *n* (%)	*p* Value
VDR rs2228530			
GG to AG	6 (20)	6 (20)	
AA to AG	2 (6.7)	4 (13.3)	
AG to GG	2 (6.7)	0 (0)	
	10 (33.4)	10 (33.4)	0.371
AG to AG	16 (53.3)	12 (40)	
GG to GG	4 (13.3)	6 (20)	
AA to AA	0 (0)	2 (6.7)	
	20 (66.7)	20 (66.7)	0.260
CYP2R1 rs10741657			
GG to AA	6 (20)	10 (33.3)	
AG to AA	2 (6.7)	0 (0)	
GG to AG	4 (13.3)	4 (13.3)	
AA to AG	6 (20)	4 (13.3)	
AG to GG	2 (6.7)	0 (0)	
AA to GG	0 (0)	2 (6.7)	
	20 (66.7)	20 (66.7)	0.212
AA to AA	2 (6.7)	0 (0)	
AG to AG	8 (26.7)	8 (26.7)	
GG to GG	0 (0)	2 (6.7)	
	10 (33.4)	10 (33.4)	0.226

VDR: vitamin D receptor; Fisher’s exact test (two-sided).

**Table 4:** Serum 25(OH)D concentrations in recipients before and after LDLT with rejection (*n* = 30) and non-rejection (*n* = 30) graft pathology.

Serum Biomarkers		Rejection *n* = 30	Non-Rejection *n* = 30	*p* Value
25(OH)D *	Before LDLT	54.33 ± 29.53 ^a^	41.56 ± 31.70 ^b^	a:a’ = 0.0001
	After LDLT	11.01 ± 7.64 ^a’^	11.32 ± 7.55 ^b’^	b:b’ = 0.0017
MELD Score before LDLT, mean ± SD		19.37 ± 3.07	20.53 ± 4.02	ns
ALT (IU/L) after LDLT		65.30 ± 17.68	69.45 ± 25.38	ns
AST (IU/L) after LDLT		49.30 ± 13.44	52.25 ± 16.23	ns

LDLT: living donor liver transplantation; MELD Score: Model For End-Stage Liver Disease Score *: ng/mL in unit; ns: no statistical significance; Student’s *t*-test with two tails, type 1 model. * The mean serum 25(OH)D level was 44.59 ± 36.93 ng/mL in an additional 30 recipients with normal controlled. This was statistically significantly higher than both rejection (11.01 ± 7.64, *p* < 0.005) and non-rejection with graft pathology groups (11.32 ± 7.55, *p* < 0.005) after LDLT.

## Data Availability

Because of the participant consent obtained as part of the recruitment process, it is not possible to make these data publicly available. The data resented in this study are available on request from the corresponding author.
